# Optical management for efficiency enhancement in hybrid organic-inorganic lead halide perovskite solar cells

**DOI:** 10.1080/14686996.2018.1458578

**Published:** 2018-05-24

**Authors:** Hui Zhang, Johann Toudert

**Affiliations:** a Key Laboratory of Flexible Electronics (KLOFE) and Institute of Advanced Materials (IAM), Jiangsu National Synergetic Innovation Center for Advanced Materials (SICAM), Nanjing Tech University (NanjingTech), Nanjing, P.R. China; b ICFO – Institut de Ciencies Fotoniques, The Barcelona Institute of Science and Technology, Barcelona, Spain

**Keywords:** Solar cells, light harvesting, perovskite, optical management, 50 Energy Materials, 209 Solar cell / Photovoltaics

## Abstract

In a few years only, solar cells using hybrid organic–inorganic lead halide perovskites as optical absorber have reached record photovoltaic energy conversion efficiencies above 20%. To reach and overcome such values, it is required to tailor both the electrical and optical properties of the device. For a given efficient device, optical optimization overtakes electrical one. Here, we provide a synthetic review of recent works reporting or proposing so-called optical management approaches for improving the efficiency of perovskite solar cells, including the use of anti-reflection coatings at the front substrate surface, the design of optical cavities integrated within the device, the incorporation of plasmonic or dielectric nanostructures into the different layers of the device and the structuration of its internal interfaces. We finally give as outlooks some insights into the less-explored management of the perovskite fluorescence and its potential for enhancing the cell efficiency.

## Graphical abstract

**Figure:**
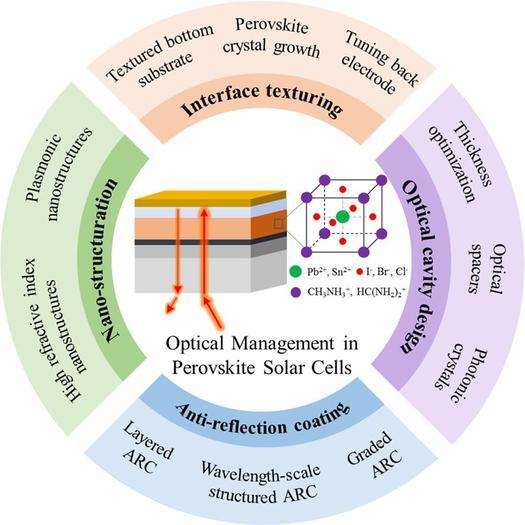

## 1. Introduction

Photovoltaic cells based on the emerging hybrid organic–inorganic lead halide perovskites (called hereafter ‘perovskite solar cells’) have attracted an increasing attention due to their excellent optoelectronic properties and low cost fabrication procedure. The rapid progress in the reported power conversion efficiency (PCE) in the last decade has surpassed everyone’s expectations. A record PCE of 22.1% has been reported recently [1]. Even if still lagging behind the theoretical limit [2], the achieved PCEs are already comparable with that of polycrystalline silicon solar cells.

In perovskite solar cells, the semiconductor perovskite material acts as an efficient optical absorber. The low binding energy of perovskite excitons ensures the efficient formation of photogenerated electrons and holes, whose bulk diffusion lengths can reach over hundreds of nano-metres [3] and are thus mostly limited by the size of the crystalline domains [4]. These photogenerated carriers can be efficiently collected by properly designed charge-selective electrodes. These features enable highly efficient light harvesting and PCEs in thin-film solar cells with sub-micrometre thickness provided the composition [5] and structure [6] of the perovskite material and the corresponding functional layers [7,8] are suitably controlled.

**Figure 1.**
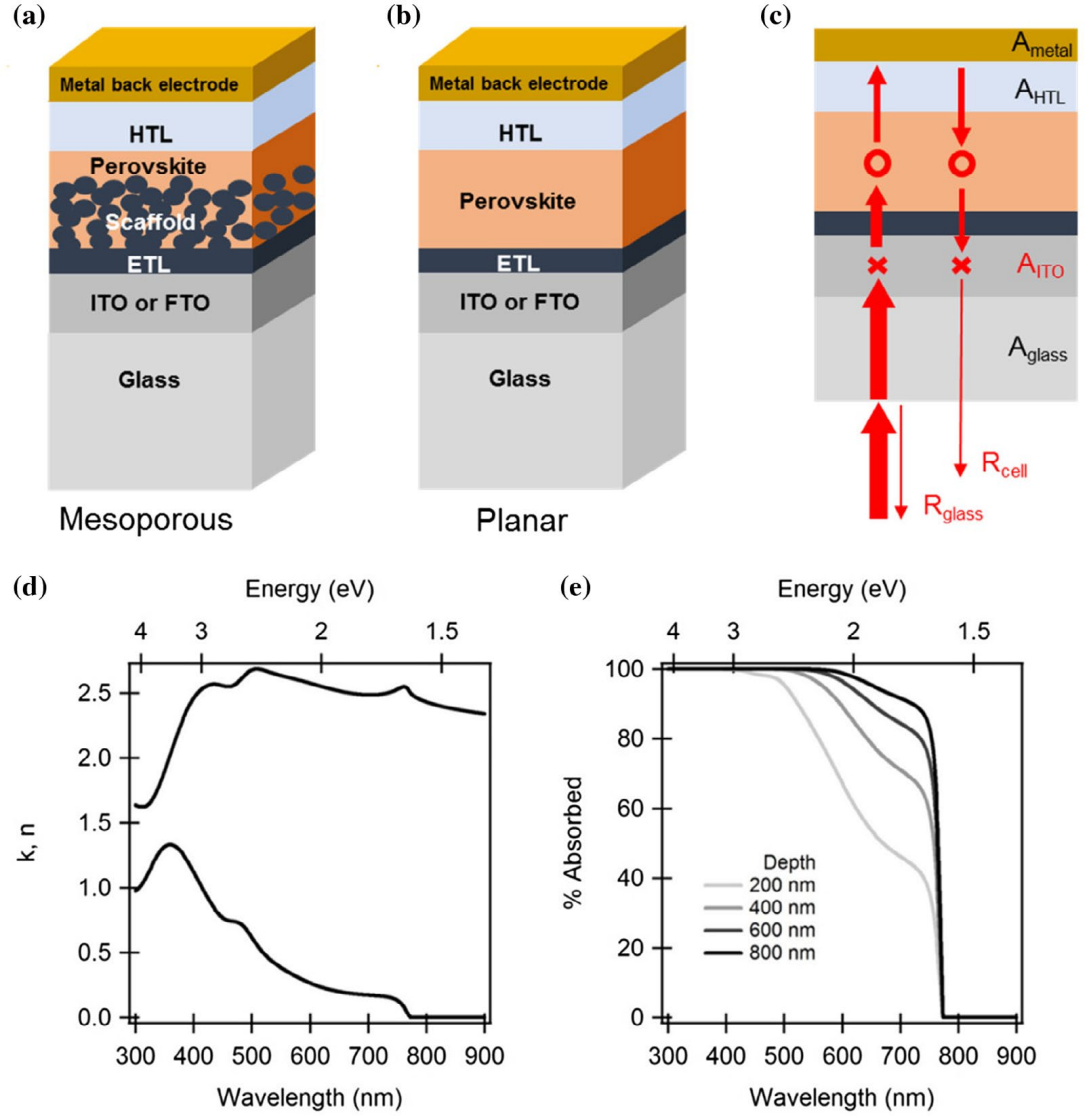
Device configuration of standard (a) mesoporous and (b) planar perovskite solar cells. (c) Schematic representation of phenomena limiting the light-harvesting efficiency in planar perovskite solar cells. (d) Optical constant *n* and *k* of a typical CH_3_NH_3_PbI_3_ perovskite. (e) Optical absorbance *A* = 1 − exp(4*πk*/*λ*) of perovskite material with different propagation depths.

As shown in Figure 1(a) and (b), the existing standard perovskite solar cells can be classified into two groups depending on their configurations: mesoporous and planar cells. In mesoporous cells, the perovskite material fills and covers a porous transparent semiconductor scaffold layer (frequently made of TiO_2_ or Al_2_O_3_) that extracts the photogenerated electrons to a compact electron transport layer (ETL, also frequently made of TiO_2_). The photogenerated holes are extracted by a hole-transport layer (HTL, for instance, made of spiro-OMeTAD) grown onto the perovskite. In planar cells, the porous scaffold layer is skipped to avoid the costly high-temperature treatment in the device fabrication process. Nowadays, both types of cells can reach efficiencies above 20% owing to the careful selection of all functional layers and optimized material preparation method. For further improving their efficiency to approach the theoretical limit, optical optimization becomes increasingly important so that it can overtake the electrical one. Especially, light-harvesting enhancement can be achieved by implementing optical management strategies to correct the weaknesses of standard cells. This can be useful for developing thinner cells while maintaining a high PCE to moderate the eco-toxicological problem arising from the heavy metal lead present in the devices.

Implementing such strategies to a solar cell relies on design optimization, on additional structuration or on the incorporation of hetero-structures. In this review, we will firstly present the absorption limit in a standard cell and explain optical strategies that can be considered to enhance the device performance. Then, we will highlight the different optical management strategies already proposed or reported for enhancing light harvesting in perovskite solar cells. We will detail the underlying optical phenomena and the gain for the photovoltaic properties of the solar cell. Another important aspect that should be taken into account for the achievement of record PCEs with perovskite solar cells deals with the optical management of their fluorescence. High-quality perovskite crystals present an intense near-bandgap radiative recombination that has recently been shown to allow photon recycling effects that can boost the photovoltaic properties of solar cells. In the end of this paper, we highlight these findings and discuss how the perovskite fluorescence relates with the solar cell’s PCEs.

## 2. Limitations to the light-harvesting efficiency in standard perovskite cells

In order to explain these limitations, we focus on the case of a planar perovskite solar cell, as represented in Figure 1(c). For instance, as calculated by Wang et al., [9] for a 350 nm thick CH_3_NH_3_PbI_3_ perovskite optical absorber (bandgap energy *E*_*g*_ = 1.55 eV), with the HTL and ETL being poly (3,4-ethylenedioxythiophene):poly(styrene sulphonic ) (PEDOT:PSS) and [6,6]-phenyl-C_61_-butyric acid-methylester (PC_61_BM), respectively, only 65% of the incident solar light is absorbed by the perovskite material. The remaining 35% loss are shared by the light that escapes from the cell *R*_cell_, mostly due to insufficient light path in the perovskite layer, the absorption losses in the indium tin oxide (ITO) layer (*A*_ITO_ = 14%) due to its non-zero extinction coefficient *k*, the reflections at the air/glass interface (*R*_glass_ = 4%), and the absorption losses in the ETL, HTL and metal back electrode (*A*_parasitic_ = 2%).

*A*_parasitic_ and *A*_ITO_ are material-related and decreasing their values would primarily require tuning the refractive index *n* and extinction coefficient *k* of these materials. An important contribution to *R*_cell_ is brought by the red and near infrared light. At wavelengths above 600 nm, the extinction coefficient *k* of the CH_3_NH_3_PbI_3_ perovskite takes smaller values than at shorter wavelengths and decreases down to near zero in the near-bandgap region (Figure 1(d)). Therefore, as supported by Figure 1(e) that shows the spectral absorbance of the perovskite as a function of propagation depth, a relevant fraction of the red and infrared light impinging at normal incidence can reach the metal back electrode where it is reflected, propagates back to the glass substrate and escapes from the *R*_cell_. This amount of unharvested light can be decreased by tuning the perovskite composition to bring its bandgap energy slightly deeper in the infrared, or by increasing the perovskite thickness to increase the optical path in the perovskite material. However, such thickness increase can be detrimental to the electrical performance of the cell, especially if additional grain boundaries are introduced in the perovskite layer during the growth. Optical management in the device architecture provides solutions complementary or alternative to the aforementioned ones, for example,

• *Anti*-*reflection coatings implemented at the air/glass interface.* They allow decreasing *R*_glass_. Many anti-reflection designs also incorporate optical scattering capabilities for focusing or increasing the optical path of light in the perovskite material, thus increasing the optical absorption in this medium (and reducing *R*_cell_).
• *Vertical optical cavity design*. It allows tuning the electromagnetic field in-depth profile in planar solar cells so that the absorption in the perovskite material and (or) the cell reflectance/transmittance are optimized.
• *Plasmonic or high refractive index nanostructures incorporated in the different layers of the cells.* Plasmonic nanostructures have the capability to scatter light, and in addition they can localize the electromagnetic energy in their surrounding region (near-field enhancement) and thus allow improving the optical absorption in perovskite material. Dielectric nanostructures are useful because of their optical scattering capability.
• *Texturing of the internal interfaces of the cell*. This texturing yields optical scattering capabilities to the considered interfaces suitable for increasing absorption in the perovskite material.

In the following, we describe in more detail these four approaches as applied to the case of perovskite solar cells.

## 3. Optical management for enhanced light harvesting in perovskite solar cells

### 3.1. Anti-reflection coatings at the air-glass interface

**Figure 2.**
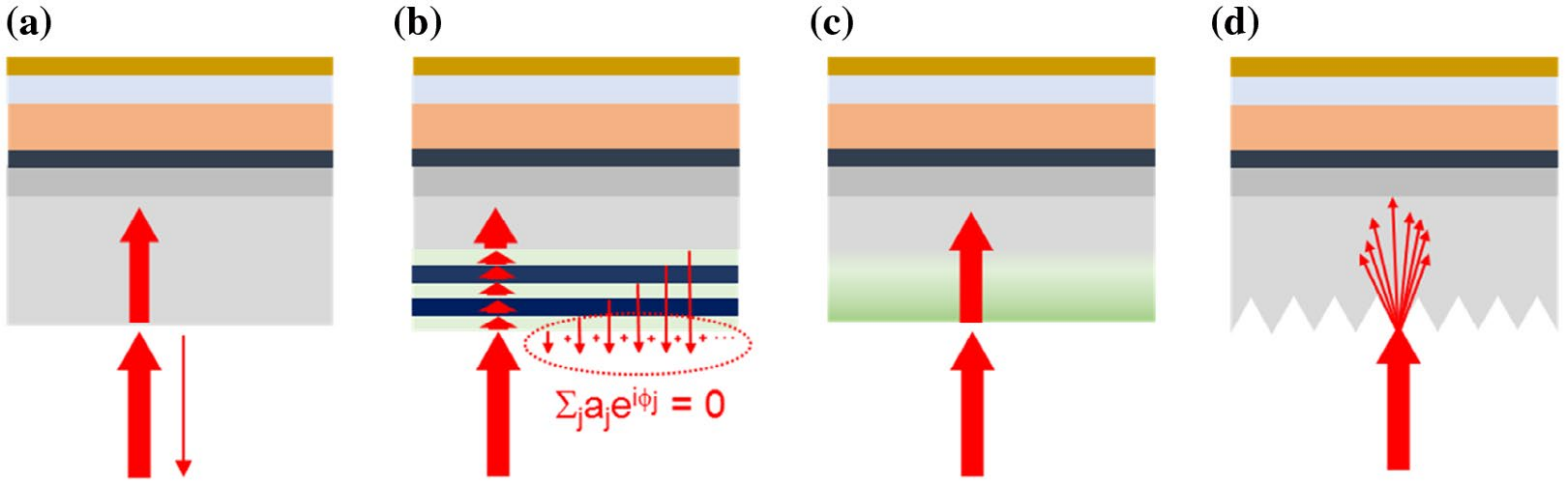
Working principle of ARCs: (a) solar cell without ARC, (b) with layered ARC (destructive interferences), (c) with graded ARC, (d) with wavelength-scale structured ARC.

For a solar cell fabricated on a planar glass substrate, 4% of the incident light at normal incidence is reflected at the air-glass interface, as depicted in Figure 2(a). This value can go up to 30% if all incident angles are taken into consideration [10]. It is known that the reflection loss can be reduced upon implementing an anti-reflection coating (ARC) on the front side of the substrate to enhance absorption in the perovskite material and thus improve the PCE of the cells.

To design and incorporate an ideal ARC for perovskite solar cells, the ARC should present a good broadband and omnidirectional efficiency across the whole wavelength region where the perovskite material absorbs, low polarization sensitivity, and protection capabilities. This last aspect is especially important for outdoor application of the solar panel, and the water accumulation on the front surface can increase the potential of performance degradation. Therefore, making the ARC hydrophobic can help protecting the device from contamination and degradation. Coherent calculations using the transfer matrix method allow designing stacks of thin transparent layers with different refractive indices to be deposited at the air/glass interface. Based on the achievement of destructive optical interferences (as depicted in Figure 2(b)) such stacks allow achieving a reduced reflectance across the entire relevant wavelength region [11]. However, because optical interferences in thin layers are sensitive to the optical path length, the properties of such ARCs depend on the angle of incidence.

Graded ARCs are in principle more prone to showing a broad angular efficiency. Rigorously speaking, such ARC consists of a stack of homogeneous ultrathin transparent layers with different refractive indices, ranging from that of the substrate (*n*_glass _≈ 1.5) to that of air (*n*_air _≈ 1.0). This removes the step difference in refractive index seen by the incident light when it impinges onto the glass and is responsible for its partial reflection (as depicted in Figure 2(c)). However, there is only few dense materials with low index and high optical transparency that can be fabricated as ultrathin layers, such as magnesium fluoride (MgF_2_, *n* = 1.39) and polydimethylsiloxane (PDMS, *n* = 1.40). Lower refractive index values can be achieved using porous materials [12]. Furthermore, metamaterial design has allowed to fabricate optically homogeneous transparent films with a refractive index down to 1.025 across the visible range [13]. However, the thickness of these ultralow index materials remains comparable with the wavelength of visible light.

In the case of perovskite solar cells, a frequently reported approach to achieve anti-reflection properties consists in introducing wavelength-scale transparent structures at the front glass surface. In short, these structures scatter the incident light toward the perovskite material, thus reducing *R*_glass_. Furthermore, this effect allows focusing light or increasing its optical path in the perovskite material. Both effects can increase the optical absorption in this medium (as depicted in Figure 2(d)), especially in the near-bandgap spectral region where it has a low extinction coefficient and thus poorly absorbs light especially at normal incidence.

The nano- and micro-structuration of ARCs can be realized using various technologies that differ by their cost and large-scale applicability such as electron-beam or optical lithography, nanoimprint, soft lithography. Using these techniques, ARCs made of many different structures have been demonstrated.

**Figure 3.**
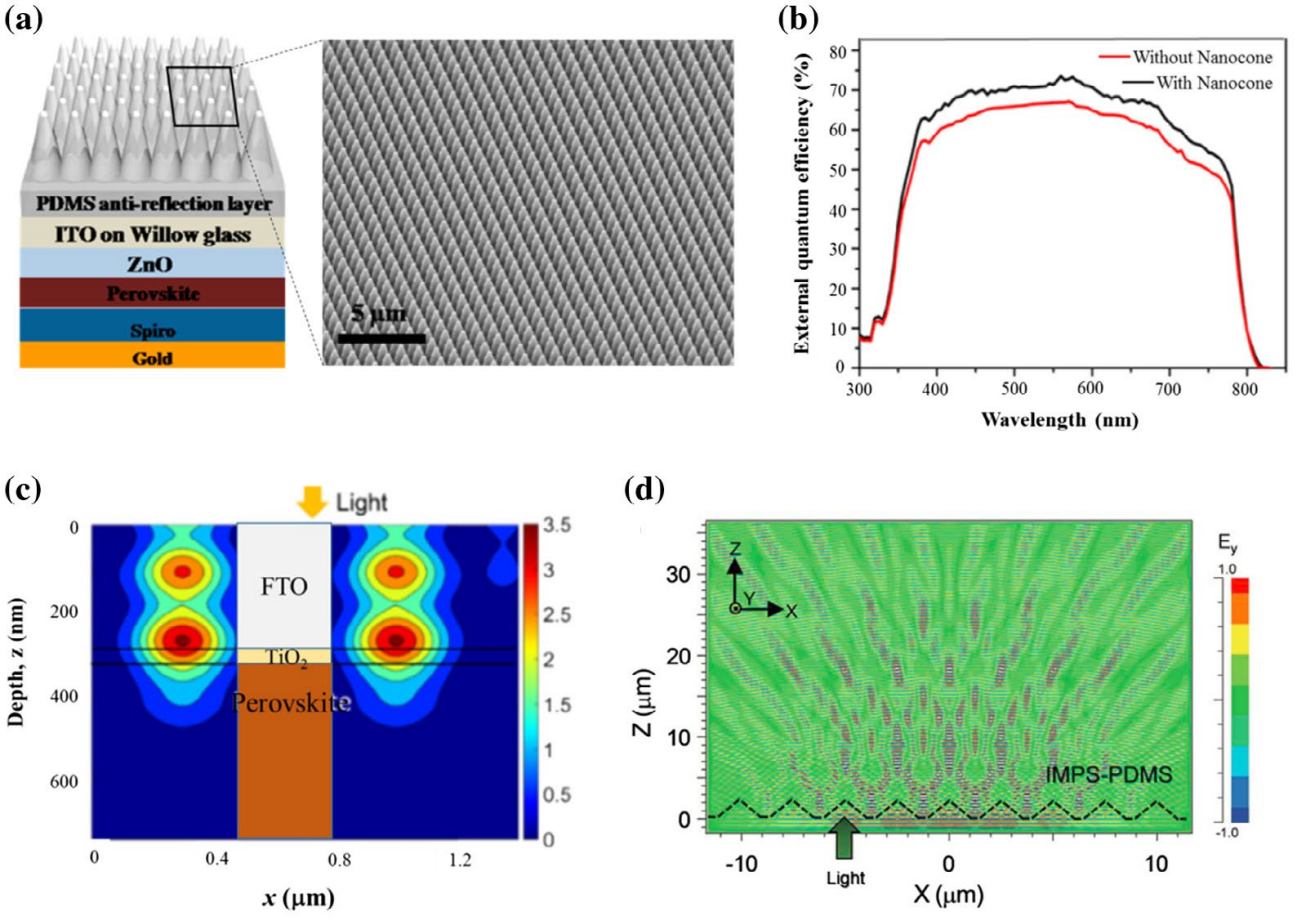
(a) Schematic representation of the solar cell structure including nanocone assembly ARC and (b) the EQE enhancement due to the reduced reflection loss. Adapted with permission from Tavakoli et al., *ACS Nano* 2015;9(10):10287. © 2015 American Chemical Society. (c) Calculation of the electric field intensity in a perovskite solar cell showing the light focusing capability of the ARC. Adapted with permission from Peer et al., *Opt. Express*. 2017;25(9):287886. © 2017 Optical Society of America. The image shows the field map inside the fluorine-doped tin oxide (FTO), TiO_2_ and perovskite layers (schematized as inset). The light impinges from the top onto the front glass surface where the ARC stands (not seen on the image). Nodes of field intensity can be seen in the FTO with their field distribution extending into the perovskite. (d) Calculation of the electric field intensity in the glass substrate of a solar cell including a micropyramid-based ARC showing the oblique propagation of light due to scattering by the surface texture (IMPS stands for inverted micro-pyramidal structure). Reproduced with permission from Dudem et al., *J. Mater. Chem. A.* 2016;4: 7573. © 2016 Royal Society of Chemistry.

For example, periodic nanostructures consisting of PDMS nanocone arrays (Figure 3(a)) were introduced as ARC at the air/glass interface of both perovskite single-junction [14] and tandem solar cells [15]. The presence of the ARC improves the EQE in the entire visible region, as shown in Figure 3(b), because the nanocone array scatters light preferentially toward the inside of the cell thus leading to a reduced reflection at the substrate surface. It also focuses light in part into the perovskite material (Figure 3(c)). The nanocone aspect ratio, defined as the ratio between their height and their width, plays a decisive role in controlling these effects. When this aspect ratio approaches to 1, the absorption enhancement is around 8% in a broad wavelength range from 400 to 850 nm. Moreover, this ARC is effective in a broad range of incidence angles; it is also hydrophobic, which can enhance the water resistance of a device and provide the function of self-cleaning by rainfall. Similar optical effects have been reported experimentally using an ARC consisting of nanodome arrays [16].

ARCs made of disordered assemblies of microscale transparent structures imprinted at the air/glass interface of perovskite solar cells have also been reported. As the nanocones, these structures scatter light toward the inside of the cell (Figure 3(d)). For the incoming light impinging onto the cell at normal incidence, the scattered waves propagate obliquely (Figure 3(d)) thus potentially increasing the optical path length in the perovskite material. Such structures can consist of inverted PDMS inverted micro-pyramids [17], microscale pyramids [18] or microscale rose petals [19].

The photovoltaic parameters of the solar cells, prior and after incorporation of the ARC, are gathered in Table 1, showing mainly an increase in the short circuit current (J_SC_), while the other parameters such as open-circuit voltage (V_OC_) and fill factor (FF) remain unchanged by the addition of the ARC at the air/glass interface.

**Table 1.** Photovoltaic parameters of perovskite solar cells with (‘ARC’) and without (‘Ref’) an anti-reflection coating placed at the air/glass interface of the cell.

Source	J_SC_		V_OC_		FF		PCE	
	(mA/cm^2^)		(V)		(%)		(%)	
	Ref	ARC	Ref	ARC	Ref	ARC	Ref	ARC
[14]	17.7	19.3	0.97	0.98	70	69	12.06	13.14
[15]	16.1	18.5	–	–	–	–	–	–
[17]	20.6	21.2	1.09	1.09	76.6	76.6	17.17	17.74
[18]	20.7	21.7	1.11	1.11	70.9	71.2	16.3	17.1
[19]	20.8	24.5	1.03	1.03	77.6	75.9	16.6	19.2

Structures coupling macroscale textures combined with Bragg reflectors have been proposed for improving the efficiency of silicon/perovskite tandem cells and to achieve up to 4% PCE enhancements compared with a planar tandem cell [20,21]. In addition to the approaches described above, some alternative device configurations render the substrate anti-reflective. For example, it has been predicted that a well-aligned and suitably designed dielectric nanofibre array used to replace the planar glass substrate, has the potential to enhance the light absorption in the perovskite material of 6.3% by minimizing the cell reflectance [22].

### 3.2. Vertical optical cavity design

**Figure 4.**
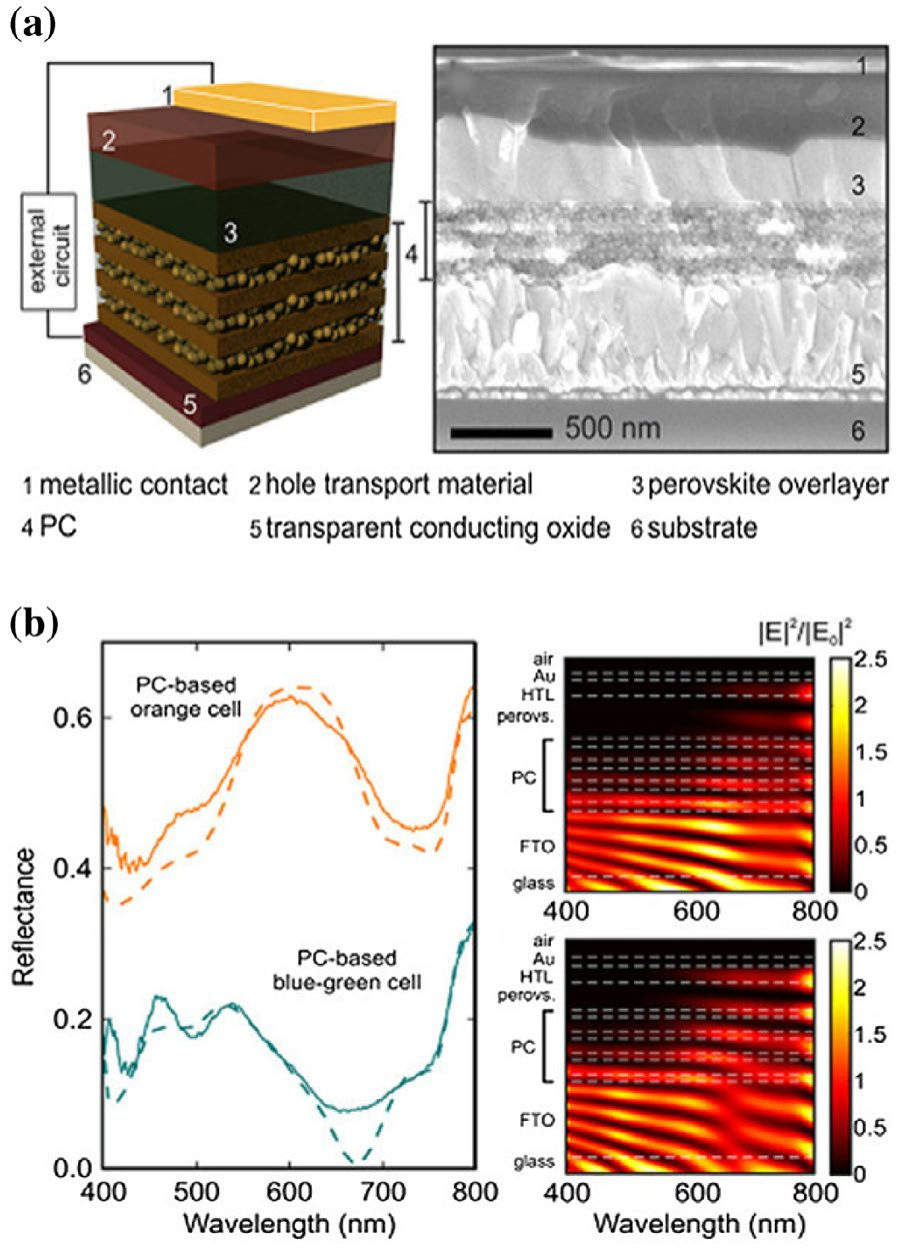
(a) Structure of a photonic structure-based cell. The ETL consists of a porous vertical photonic structure (PC stands for photonic crystal) alternating SiO_2_:TiO_2_ and TiO_2_ layers (b). Spectra of the reflectance (solid lines: measurements, dashed lines: calculations; vertically offset for clarity) and calculated wavelength-dependent in-depth distribution of the electric field intensity in the solar cell for two different configurations of the vertical photonic structure (different thicknesses for the SiO_2_:TiO_2_ and TiO_2_ layers). Changing this configuration changes the reflectance spectrum and thus the colour of the cell (in this figure, one has an orange aspect, the other one a blue-green aspect), as well as the in-depth distribution of the electric field. Reproduced with permission from Zhang et al., *Nano Lett.* 2015;15: 1698. © 2015 American Chemical Society.

In a solar cell, reflection of the incident light occurs at each interface where there is a contrast in optical constants. In the thin layers constituting the cell, interferences between monochromatic waves propagating forward and backward dictate the in-depth profile of the electromagnetic field [23]. This phenomenon is useful for tuning the optical absorption in the different layers and the reflectance/transmittance of the cell in specific spectral regions. Such tuning can be realized by adjusting the thickness of the different layers in a standard perovskite solar cell, or by introducing additional layers with suitable optical properties to the structure as optical spacers. The best configurations can be determined from numerical simulations based for instance on the transfer matrix-method [23,24] or full-wave methods [25]. There have been several demonstrations of vertical optical cavity design for achieving perovskite solar cells with tailored optical properties or optimized photovoltaic parameters. For instance, the reflectance [26,27] and transmittance [28] of perovskite solar cells with low-to-moderate PCEs have been tuned through the entire visible region to control their apparent colour, either by tuning the layer thicknesses in a SiO_2_:TiO_2_/SiO_2_ 1D photonic crystal ETL, the thickness of a WO_3_ layer incorporated between the perovskite material and the metal back electrode, or the thickness of a PEDOT:PSS back electrode. The effect of implementing a SiO_2_:TiO_2_/SiO_2_ 1D photonic crystal ETL is shown as example in Figure 4. By tuning the thickness of the different layers of the photonic crystal, the optical phase shift between the partially reflected waves in the cell, and thus the interference between them, can be controlled. This allows tuning the in-depth distribution of the electromagnetic field in the different layers (Figure 4(b), right) and the reflectance spectrum of the cell (Figure 4(b), left). By such means, the cell colour can be tuned from orange to blue-green. More recently, it has been proposed that a two-resonance tapping cavity design could allow nearly matching the external quantum efficiency (EQE) to the internal one (IQE) in perovskite solar cells with a perovskite optical absorber layer thicker than 600 nm [23]. Finally, a simpler application of vertical optical design consists in developing efficient anti-reflection coating structures at the air/glass interface of the solar cell. This was the subject of Section 3.1.

### 3.3. Nanostructures incorporated in the different layers of the cell

#### 3.3.1. Plasmonic nanostructures

**Figure 5.**
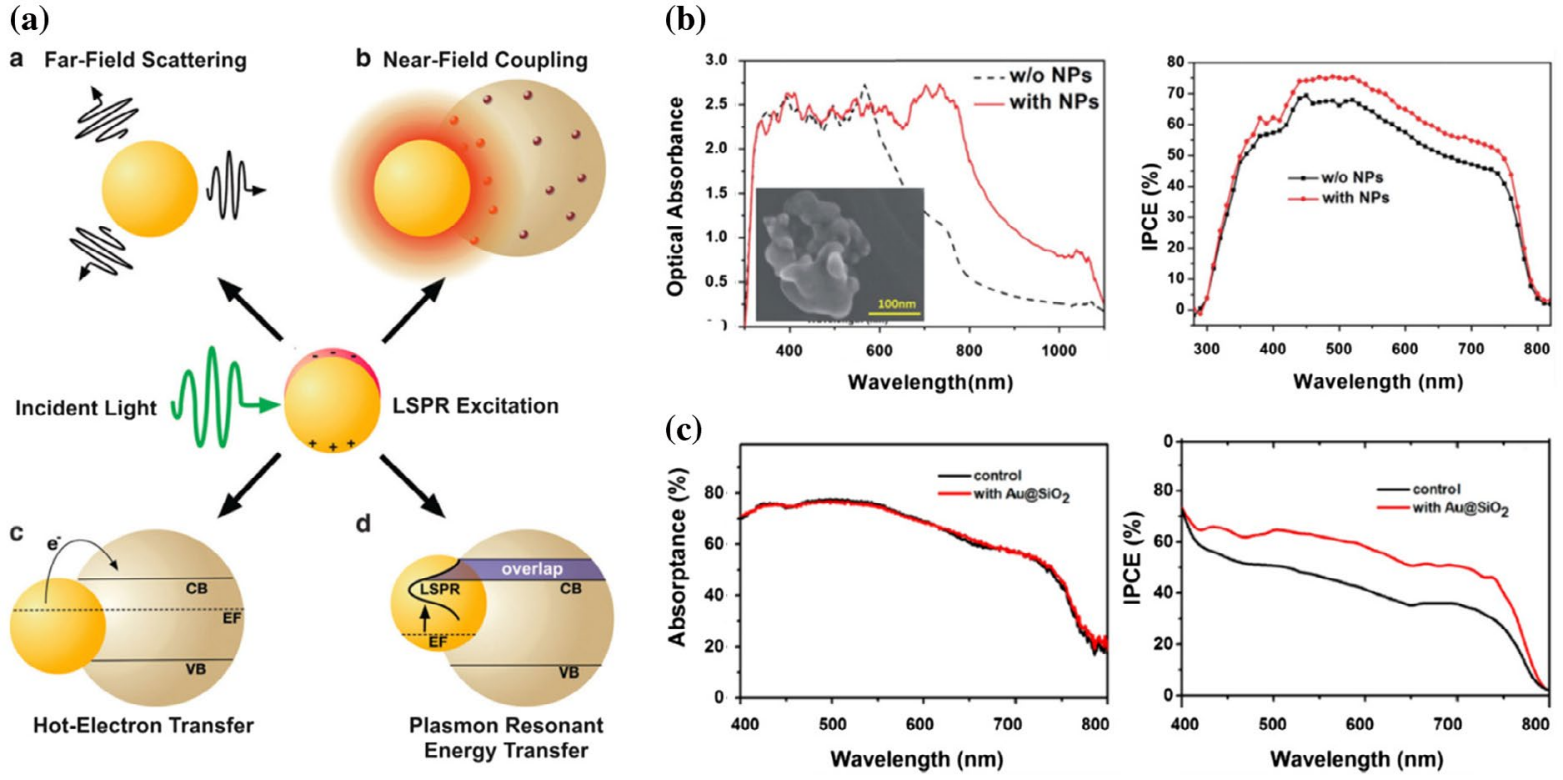
(a) Schematic of plasmonic effect in perovskite solar cells **a**: far-field scattering, **b**: near-field coupling, **c**: hot-electron transfer and **d:** plasmon resonant energy transfer. Reproduced with permission from Erwin et al., *Energy Environ, Sci.* 2016;9:1577. © 2016 Royal Society of Chemistry. (b) Optical absorbance of a mesoporous perovskite solar cell with and without popcorn-shaped Au –Ag nanoparticles, and corresponding EQE spectra. Reproduced with permission from Lu et al., *RSC Advances.* 2015;5:11175. © 2015 Royal Society of Chemistry. (c) Absorptance of the perovskite layer in Al_2_O_3_-based device without (control) and with Au@SiO2 NPs and corresponding incident photon-to-electron conversion efficiency (IPCE) spectra. Reproduced with permission from Zhang et al., *Nano Lett.* 2013;13:4505. © 2013 American Chemical Society.

The interest in plasmonic nanostructures (such as nanoparticles, nanorods, nanoshells, nanostars) comes from their capability to support the so-called localized surface plasmon resonances (LSPRs). This effect results from the association of the electromagnetic field of the incident light with the free electrons in the nanostructure (frequently made of a metal), which induces an electromagnetic resonance [29]. The LSPR induces a strong surface polarization of the nanostructure, which can thus lead to a strong enhancement of the electromagnetic field at its nanoscale vicinity (‘near-field enhancement’) and radiate electromagnetic waves (‘scattering’ to the far field), as depicted in Figure 5(a). These effects can be useful for increasing absorption in the optical absorber layer of a solar cell, by localizing the LSPR near-field or increasing the optical path length in it, respectively. However, to make these enhancements effective, it is crucial to properly choose the nature, size, shape and localization of the nanostructures in the devices, because these parameters affect strongly their near-field and scattering properties that compete with the optical absorption by the metal, as well as the spectral position of their LSPRs [30].

Besides their potential for enhanced light trapping using the radiative effects cited above, plasmonic nanostructures can also have a positive impact through non-radiative effects (Figure 5(a)). Plasmons can transfer their energy non-radiatively to the surrounding semiconductor medium following the so-called ‘plasmon resonant energy transfer’ process. Moreover, after the femtosecond plasmon decay into electron-hole pairs, the so-called ‘hot electrons’ can be extracted into the surrounding semiconductor medium.

Plasmonic nanostructures with different nature, size, shape and concentration have been incorporated into the different layers of perovskite solar cells. Several works (Table 2) have reported the incorporation of plasmonic nanostructures in the scaffold layer of mesoporous perovskite solar cells: 80 nm Au@SiO_2_ spherical core –shell nanoparticles in an Al_2_O_3_ scaffold [31], 40 nm Ag@TiO_2_ spherical core –shell nanoparticles in an Al_2_O_3_ scaffold [32], Au@SiO_2_ spherical nanoparticles and nanorods with 40 nm equivalent diameter in an Al_2_O_3_ scaffold [33], 100 nm Au –Ag alloyed popcorn-shaped nanoparticles in a TiO_2_ scaffold [34], an Au nanoparticles (60 nm)/TiO_2_ nanofibre scaffold [35] as well as Au nanostars in a TiO_2_ scaffold [36]. Encapsulating the metal into a dielectric shell allows avoiding parasitic recombination of the photogenerated carriers at the interface of metal/perovskite, and a chemical reaction between the metal and the perovskite that can be detrimental to the device performance. Making the plasmonic nanostructures aspherical allows shifting their LSPR from wavelengths below 600 nm (resonance of a spherical Au or Ag nanoparticle) to the near infrared (nanorods), or to achieve a distribution of plasmonic modes across the visible (nanostars and popcorn-shaped nanoparticles). Such a morphology control is essential so that the plasmonic nanostructures enhance the optical absorption in the red and infrared region where the perovskite material does not absorb light efficiently [34] (Figure 5(b)).

**Table 2.** Photovoltaic parameters of perovskite solar cells with the same fabrication parameters, with (‘NSs’) embedded plasmonic nanostructures, and without them (‘Ref’). The data have been taken from the corresponding references in the text.

Source	NSs	J_SC_ (mA/cm^2^)		V_OC_ (V)		FF (%)		PCE (%)		Effect
		Ref	NSs	Ref	NSs	Ref	NSs	Ref	NSs	
[31]	Au@SiO_2_	14.8	16.9	1.02	1.04	64	67	10.7	11.4	Reduced exciton energy
	80 nm spheres									
	0.9 wt%									
[32]	Ag@TiO_2_	17.3	19.7	1.03	1.04	64	67	11.4	13.7	Photon recycling
	40 nm spheres									
	2.2 wt%									
[33]	Au@SiO_2_	13.9	17.4	1.17	1.16	66	68	10.7	13.7	Enhanced absorption
	40 nm rods									Better charge collection
	2.0 wt%									
[34]	Au –Ag	15.5	16.5	0.92	0.95	63	66	8.9	10.3	Enhanced absorption
	100 nm popcorn									Better charge collection
	0.7 wt%									
[35]	Au/TiO_2_	19.6	20.8	0.85	0.99	62	70	10.3	14.4	Enhanced absorption
	Fibres									Better charge collection
	0.3 wt%									
[36]	Au stars	21.1	23	1.05	1.08	69	71	15.2	17.7	Enhanced absorption
										Better charge collection
	20 nm									

In these works, the nanostructure concentration remained very low (less than a few wt% of metal) and the nanostructures were smaller than 100 nm. Despite that, the incorporation of the plasmonic nanostructures improved the PCE of the solar cells. This improvement involves in all the cases an increase in J_SC_. In most cases, there is no clear correlation between the LSPR spectrum of the individual nanostructures and optical absorption in perovskite layer of the solar cell. The improvement in PCE has thus been ascribed to the combination of an enhanced EQE spectrum and a better photocarrier collection, both effects being related to the presence of the plasmonic nanostructures. In contrast, Zhang et al., [31] found PCE improvements beyond absorption enhancement. They ascribed them to a reduction in the exciton binding energy induced by the enhanced near-field of the plasmonic nanostructures (Figure 5(c)). Furthermore, in Ref. [32], it is proposed that the plasmonic nanostructures orient the fluorescence pattern of the excitons toward the perovskite material thus inducing a ‘photon recycling’ effect.

Plasmonic nanostructures have also been embedded into the TiO_2_ ETL of planar perovskite solar cells. Ag, Au and Au –Ag alloy nanostructures with different sizes and morphology [37] allowed enhancing J_SC_ and PCEs. The highest enhancement was observed for Au –Ag nanostructures whose LSPR peaks at wavelengths above 600 nm and the authors proposed that this could be due to a scattering-induced increased optical path length in the perovskite material. In another work, increases in V_OC_, J_SC_, FF and PCE upon incorporation of spherical Au nanostructures into a TiO_2_ ETL have been attributed to hot-electron injection from the metal to the semiconductor [38].

Moreover, 20 nm Au nanoparticles have also been embedded into the HTL (spiro-OMeTAD) at the back of the cell [39]. In this work, the Au nanoparticles combined positive and negative effects on the cell performance. On the one hand, they allow a near-field optical absorption enhancement and a reduction in the HTL resistance, which contributed to the increase in EQE and J_SC_. On the other hand, the accumulation of Au nanoparticles at the HTL/perovskite interface builds up an energy barrier for charge transport and hence reduces the V_OC_. Therefore, an increased J_SC_ together with a constant FF and a slightly decreased V_OC_ induced a slight improvement in PCE (from 12.66 to 12.74%).

In summary, the incorporation of plasmonic nanostructures into perovskite solar cells can affect not only the light absorption by the cell, but also impact positively or negatively their electrical properties. Before concluding this section about the effect of plasmonic nanostructures on the performance of perovskite solar cells, let us note that numerous theoretical works have been reported to set the optimum nature, size, shape and organization of such nanostructures. It has been pointed out that particularly relevant improvements in the optical absorption by the perovskite material could be achieved by embedding core –shell spherical nanoparticles with diameters above 200 nm in the perovskite. This is required to minimize the optical absorption in the nanoparticles that is particularly strong at smaller sizes, and thus fully profit from their radiative and near-field properties [40]. Another theoretical work, however dedicated to dye-sensitized solar cells, has shown that even larger metal nanoparticles placed after the HTL at the back of the cell can enhance light absorption in the absorber layer near the Yablonovitch limit [41].

#### 3.3.2. High-refractive index nanostructures

High-refractive index (HRI) dielectric nanostructures, made of a transparent material, have the capability of scattering light due to the refractive index contrast with the surrounding material. They take advantage over plasmonic nanostructures due to their much lower (near zero) absorption of light. The scattering efficiency, which is particularly high at the nanostructure’s HRI Mie resonances [42], and the scattering angular pattern of such structures depends on their size, shape and dielectric environment. Therefore, properly designed and located HRI nanostructures can increase the optical path length of light in a solar cell and thus increase the optical absorption in the optical absorber layer.

**Figure 6.**
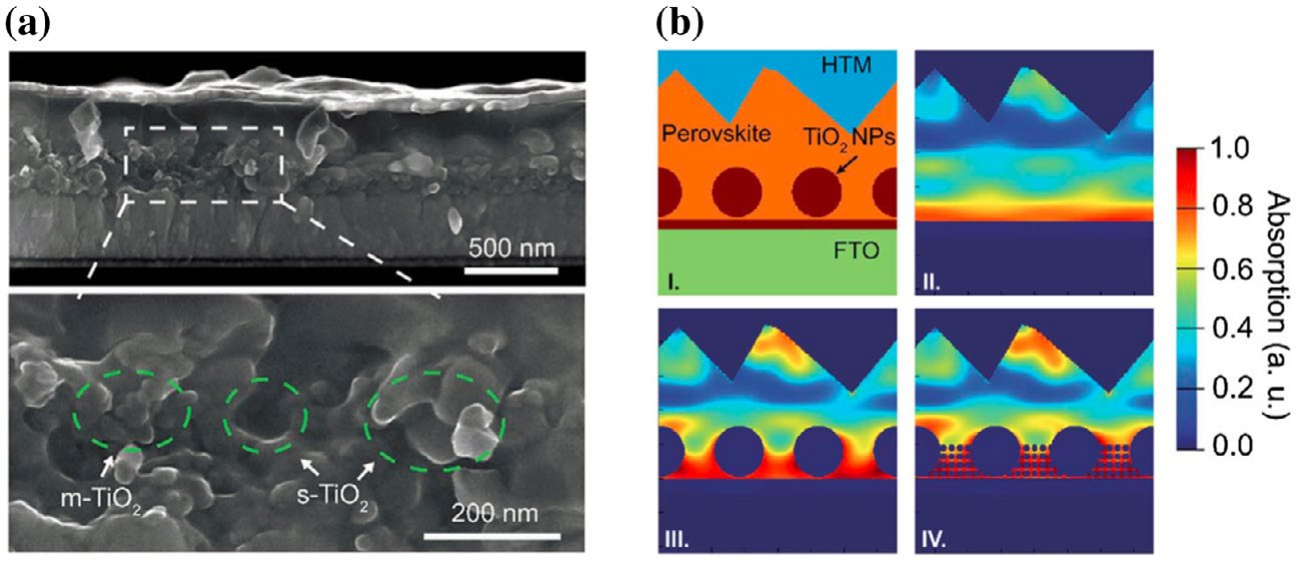
(a) Cross-sectional scanning electron microscopy (SEM) image of perovskite solar cells with incorporated mesoporous (m-TiO_2_) and sub-micron TiO_2_ (s-TiO_2_) as scaffold layer. (b) finite-difference time-domain (FDTD) simulation model (I) and (II–IV) correspondingly simulated absorption profiles (at wavelength of 600 nm) for structured perovskite devices embedded with the m-TiO_2_, s-TiO_2_ and m&s-TiO_2_, respectively. Adapted with permission from Yin et al., *RSC Advances* 2016;6:24596. © 2016 Royal Society of Chemistry.

The introduction of light scattering HRI nanostructures, frequently made of TiO_2_, has been reported in the scaffold layer of mesoporous perovskite solar cells. Huang et al., [43] and Yin et al., [44] have tuned the size of these nanostructures from a few tens of nanometres to a few hundreds of nanometres . A relative gain in PCE of 5–12% was obtained by embedding such nanostructures. For example, by incorporating some sub-micron TiO_2_ particles into the mesoporous scaffold layer (Figure 6(a)), a significant absorption enhancement can be achieved by the scattering effect (Figure 6(b)).

Although not necessarily focused on the control of light scattering, other works that show the broad tuning of the TiO_2_ nanostructure shape and size have to be underlined. Nanohelices [45], nanotubes [46], nanorods [47], nanobeads [48] and nanocolumns [49] have been successfully embedded in the scaffold layer of mesoporous perovskite solar cells. The achievable broad control in the shape of the TiO_2_ nanostructures can open the way to fine-tuning their light scattering properties. Furthermore, as for plasmonic nanostructures, note that the tuning of the scaffold layer morphology can help not only to improve the J_SC_ of the cell thanks to light scattering. It can also help reducing the V_OC_ loss and hysteresis problem owing to the increased charge transport between the ETL and perovskite.

### 3.4. Texturing of the internal interfaces of the cell

#### 3.4.1. Substrate and ETL

The introduction of dielectric nanostructures to the scaffold layer described above, beyond inducing light scattering, can also affect the morphology of the upper perovskite layer [50]. In fact, efficient light scattering can develop at the interface between these two layers, or between the ETL and perovskite layer in planar cells, if it presents a wavelength-scale texture. In this context, simulations have shown that significant optical absorption enhancement in the perovskite material can be achieved by growing the ETL and perovskite layers on a substrate with a periodic nanoscale pyramidal texture [51]. This texture allows focusing the incoming light into the perovskite layer. Another study reported the fabrication of a planar perovskite solar cell on flat and nanoscale randomly textured FTO/glass substrate [52]. In this latter cell configuration, the incoming light is scattered at the ETL/perovskite interface, and distorted vertical cavity modes establish in the perovskite layer thus inducing nodes of enhanced optical absorption. The cell fabricated on a textured FTO/glass substrate shows higher J_SC_, FF and PCE (19.8 mA/cm^2^, 63 and 13.3%) than that fabricated on a flat substrate (17.3 mA/cm^2^, 59 and 10.9%).

**Figure 7.**
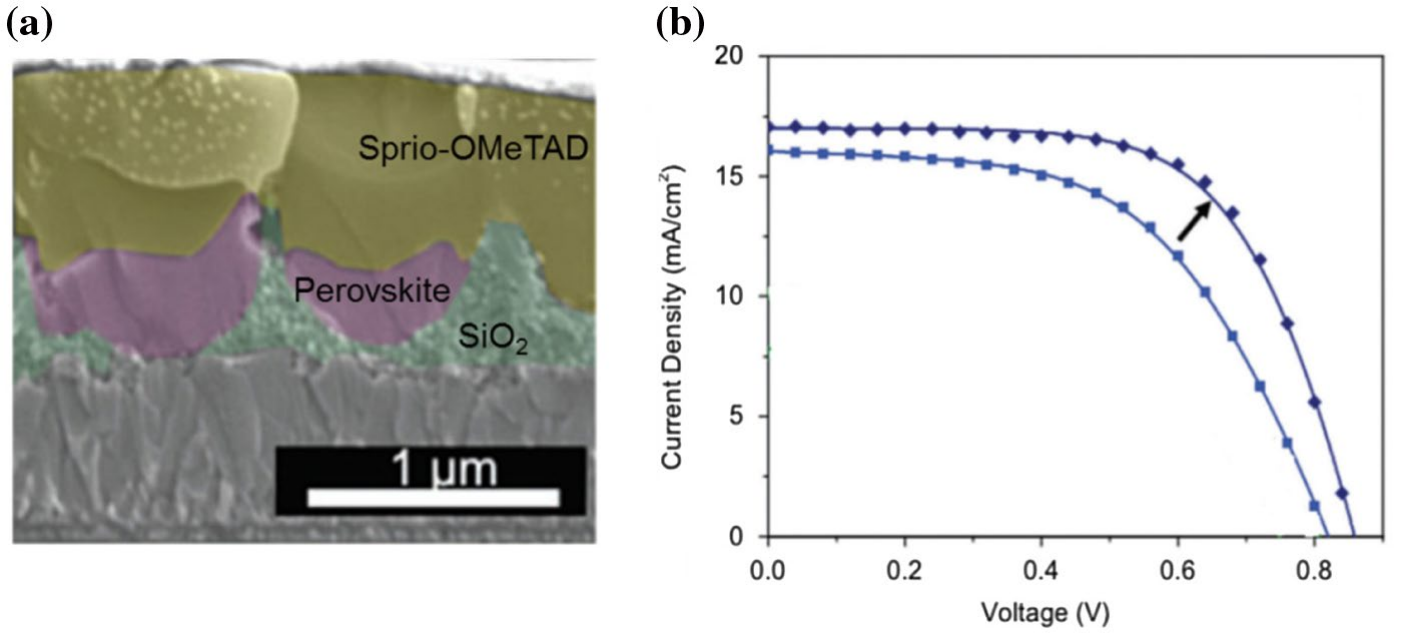
(a) Cross-sectional SEM of a perovskite solar cell fabricated on honeycomb structured SiO_2_ and (b) the J-V enhancement compared with that on planar substrate. Adapted with permission from Horantner et al., *Energy Environ. Sci.* 2015;8:2041. © 2015 Royal Society of Chemistry.

Horantner et al., [53] reported a colloidal monolayer lithography method to deposit micro-structured SiO_2_ honeycomb layer on the substrate as seen in Figure 7(a). The as-prepared substrate was then used as a template for the morphology transformation into perovskite thin films. The pore size between the spheres and periodicity of the so-prepared template can be fine-tuned by changing the size of colloidal particles and concentration of the filling material, SiO_2_. This new method can be incorporated into perovskite solar cells to enhance the light scattering and reduce the reflection of incident light. As they also found, the inserting a non-conductive SiO_2_ to separate perovskite and TiO_2_ can improve the V_OC_ and FF of the device (Figure 7(b)) due to the reduced shunting paths between HTL and ETL.

#### 3.4.2. Tuning the crystal growth

Light scattering can occur at the interfaces of perovskite crystals in a solar cell and thus enhance light absorption in the perovskite material. The solution processes which are broadly used for the fabrication of perovskite solar cells offer the possibility of tuning the size, shape, organization and orientation of the perovskite crystals [54,55], which can be particularly useful for controlling their interaction with light.

**Figure 8.**
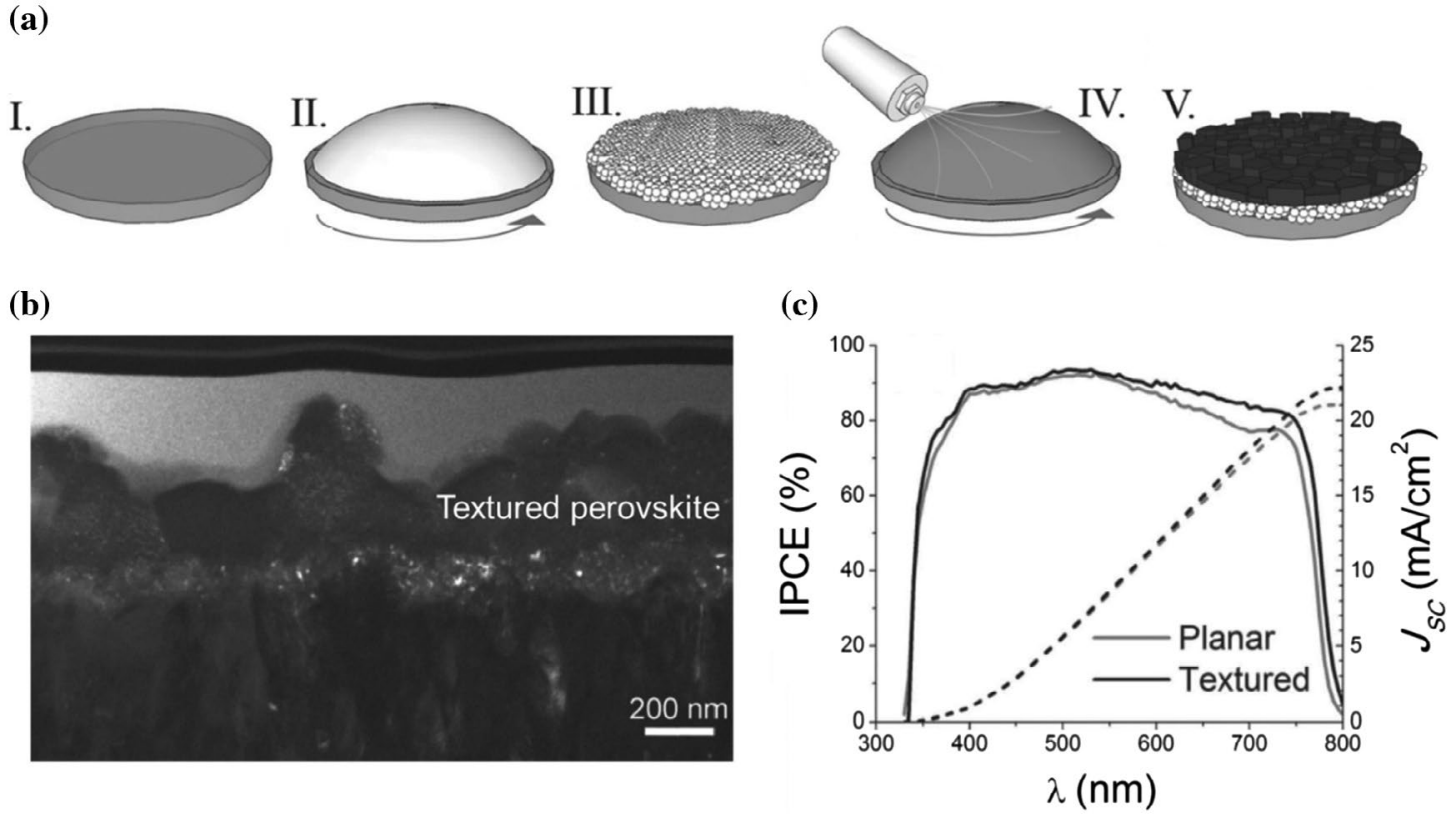
(a) Schematic illustration of the fabrication procedure: (I) A compact and (II) mesoporous TiO_2_ layer was subsequently deposited on the cleaned glass substrate, and (III) sintered to produce a dense/mesoporous bilayer film. (IV) A CH_3_NH_3_PbI_3_ precursor solution was spin-coated onto the substrate and a nitrogen gas stream was introduced to rapidly remove the solvent. (V) The film was finally annealed in an inert atmosphere, and (b) cross-sectional transmission electron microscopy (TEM) image of the textured perovskite. (c) IPCE spectrum (solid lines) of a planar perovskite device (grey line) and a textured perovskite device (black line). Adapted with permission from Pascoe et al., *Adv. Funct. Mater*. 2016;26:1278. © 2016 WILEY-VCH Verlag GmbH & Co. KGaA, Weinheim.

According to the classical LarMer model, the kinetic competition between the nucleation and crystal growth determines the final crystal surface morphology and intrinsic crystallinity. It has to be mentioned that the nucleation step, which takes place in a short time once the precursor reaches the super-saturation concentration, plays a decisive role in the final surface morphology. In order to control the perovskite nucleation process, Pascoe el al. [56] reported a gas-assisted fabrication method. A stream of nitrogen gas was introduced to accelerate the solvent evaporation during the spin coating as indicated in Figure 8(a). In the meantime, the precursor concentration and the morphology of the beneath supporting layer were fine-tuned to control the nucleation rate. It was found that two different types of nucleation occurred during the perovskite formation. A fast heterogeneous nucleation process firstly appeared on the mesoporous supporting layer, and followed by a secondary nucleation on the top. The heterogeneous nucleation resulted in a dense layer on the bottom for a good coverage and the secondary nucleation was key to the formation of textured perovskite surface (Figure 8(b)). Comparing with devices fabricated on planar perovskite, a significant enhancement in EQE was found in textured perovskite cells as shown in Figure 8(c). This enhancement was attributed to the prolonged optical path length in the perovskite layer due to the light scattering on the textured surface and voids in the non-homogenous films.

**Figure 9.**
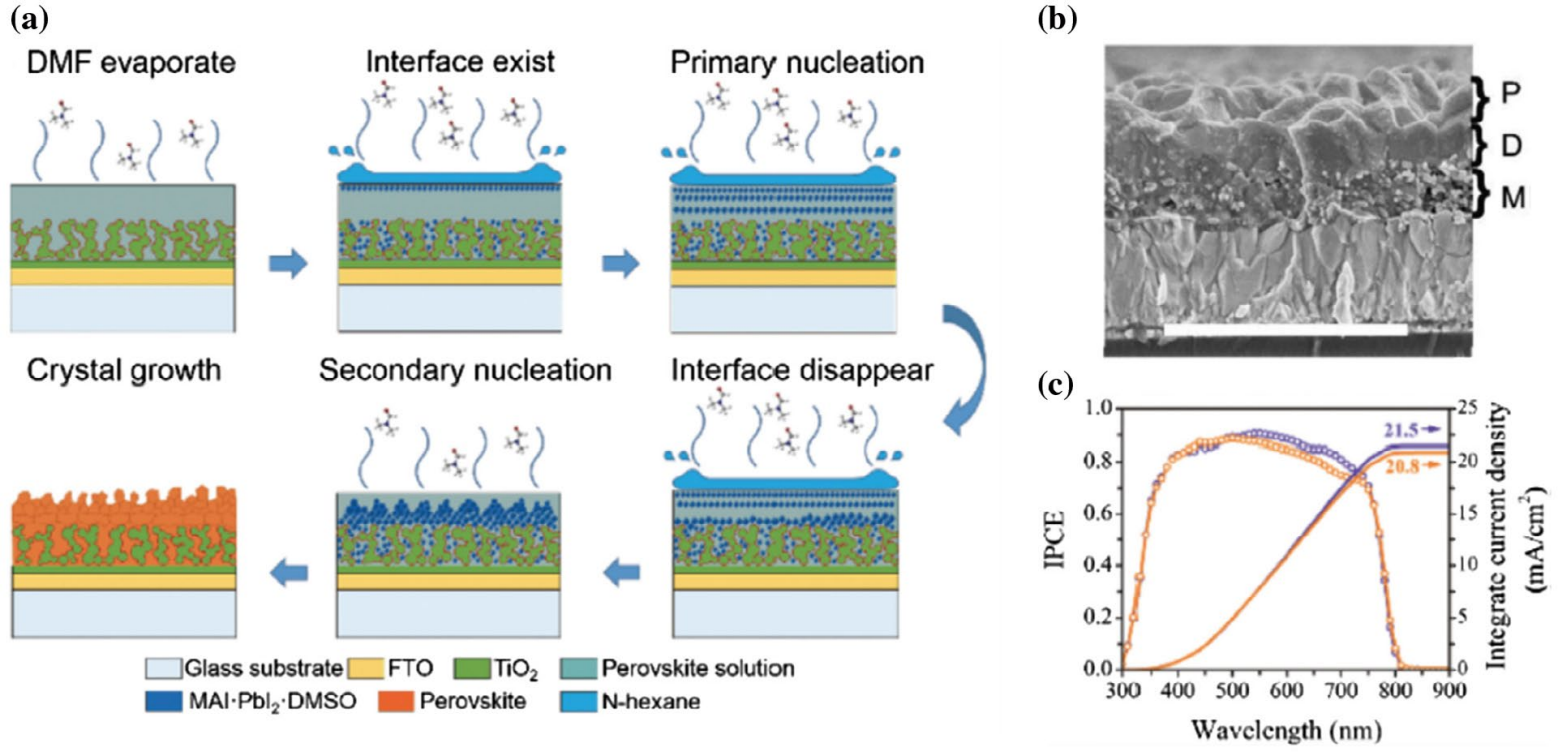
(a) Schematic illustration of nucleation and crystal growth in the formation of perovskite film with porous upper layer and (b) the cross-sectional SEM of the as formed film, (c) EQE enhancement of the device with porous upper layer (purple) compared with flat perovskite (orange). Adapted with permission from Yu et al., *Nanoscale* 2017;9:2569. © 2017 Royal Society of Chemistry.

A novel method was developed by Yu et al., [57] through solvent engineering, in which three steps sequential nucleation were involved in the perovskite formation. In this method, an additional solvent dimethyl sulfoxide (DMSO) was introduced into the precursor solution and a lower volatile solvent n-hexane was used in the anti-solvent dripping. During the spin coating, the precursor solvent dimethylformamide (DMF) started to escape from the film and an anti-solvent was then dripped on the top. Due to the low volatility of the anti-solvent, an interface between the precursor layer and the anti-solvent can be generated. Homogeneous nucleation can happen at this interface, and then heterogeneous nucleation at the bottom porous TiO_2_ and conventional secondary nucleation on the top started sequentially during the crystallization (Figure 9(a)). In the end, perovskite film consists of three layers: a mesoscopic layer on porous TiO_2_, a dense layer in the middle and a porous upper layer as shown in Figure 9(b). The morphology of the porous upper layer originated from the secondary nucleation, which can be adjusted by changing the nucleation starting time and duration via controlling the anti-solvent dripping. The porous top surface was found to enhance light scattering in the photovoltaic device, which achieved a relative 8.2% enhancement on performance compared to the device fabricated on flat perovskite (Figure 9(c)).

The surface energy of the seeding layer is another parameter that affects perovskite films growth. The substrate can be patterned with a strong contrast in surface energy by coating some functional molecules [58]. The growth of perovskite on such substrate can follow the as patterned structures according to the difference in the interaction between the perovskite and the coated molecules. Using this method, the dimension of the structure is hard to be controlled at dimensions comparable with the wavelength of visible light. In addition, the film is not continuous due to the absent of perovskite materials at hydrophobic regions. This method can find applications in some specific devices but it is not suitable for solar cells.

However, to achieve high degree of texturation on perovskite surface, it is very likely to induce some intrinsic and interfacial defect states such as pin holes, voids, grain boundaries and insufficient coverage owing to the non-uniform morphology. These defect states can act as recombination centres for photogenerated carriers and therefore decrease the V_OC_ of the textured device. Some post-treatments on the perovskite film should be taken to restrict these recombination channels, for example, chemical treatment [59,60] or post-annealing for recrystallization [61] to reduce surface and intrinsic defects in the perovskite film and hence V_OC_ loss.

#### 3.4.3. Tuning the back electrode

Besides the perovskite layer, ETL and scaffold layer, the interface between the HTL and the metal back electrodes can also be nanostructured for an enhanced optical absorption in the perovskite material [62]. Such approach has been used in the past for dye-sensitized solar cells where it yielded increased absorption in the optical absorber material due to the combined effect of plasmonic near-field enhancement and scattering-induced guided cavity modes [63].

**Figure 10.**
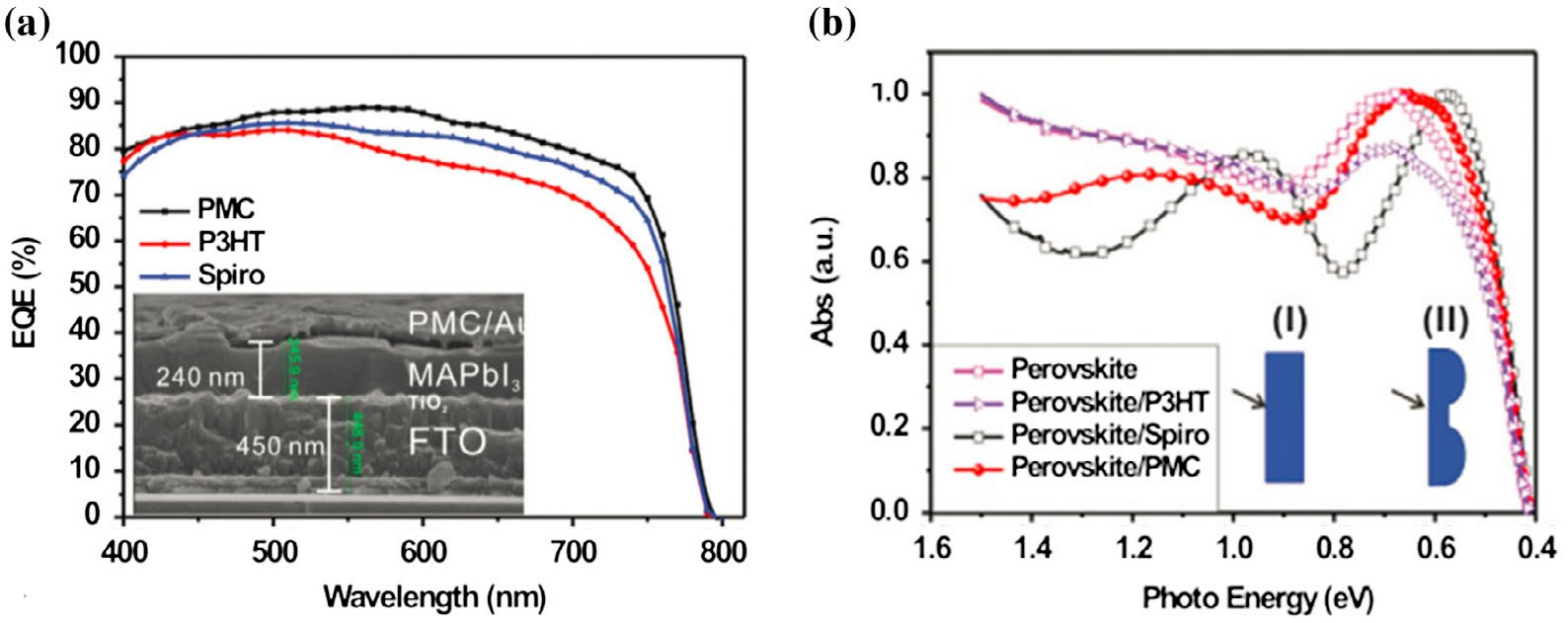
(a) EQE spectra of perovskite solar cells fabricated with different HTLs: P3HT, spiro-OMeTAD and spontaneously nanostructured P3HT/spiro-OMeTAD composite (‘PMC’). (b) Corresponding optical absorption spectra. Adapted with permission from Long et al., *Nanoscale* 2016;8:6290. © 2015 Royal Society of Chemistry.

To introduce a nanostructuration at the HTL/metal back electrode interface, Long et al., [64] replaced the spiro-OMeTAD HTL that tends to have a very flat surface [65] by a spiro-OMeTAD/P3HT composite HTL that spontaneously adopts a periodic surface nanostructure. The Au material subsequently deposited onto this layer covers it conformally. By this way, the morphology of the HTL/Au back electrode interface reproduces that of the HTL surface. The authors reported enhanced properties for the solar cell fabricated using such composite HTL compared with a reference with a standard HTL, and attributed this enhancement to improved optical absorption and electronic properties (Figure 10). The PCE was increased to 17.7% due to an increased J_SC_ and hindered hysteresis. Note that, the 17.7% PCE obtained with the aid of such composite HTL and plasmonic electrode was achieved by employing only a 240 nm thick perovskite layer. This is the highest efficiency reported using such ultrathin perovskite layer.

Using a soft imprinting technique, Wei et al., [66] introduced a bioinspired moth-eye and a grating nanostructuration at the HTL surface that was then replicated to the HTL/metal back electrode interface to promote the light harvesting in the perovskite material. Compared to the reference cell without such nanostructuration, a 14.3% improvement in J_SC_ was obtained for the moth-eye nanostructured devices, resulting in a highest PCE of 16.3%. From their experimental and theoretical characterizations, the authors propose that the cell performance enhancement is mainly ascribed to a perturbation of the Fabry –Perot cavity modes inducing hot spots of electromagnetic field in the perovskite material.

## 4. Conclusions and outlook

In summary, many approaches involving optical management have already been used to improve the performance of perovskite solar cells. They include the use of anti-reflection coatings at the air/glass interface of the cell, the tuning of the vertical configuration of the cell, the incorporation of plasmonic or dielectric nanostructures into the different layers of the cell and the structuration of the internal interfaces of the cell at the wavelength scale. However, most of the reports have shown improvements of cells with low or moderate efficiency.

While theoretical works showed the way to better take advantage of the different optical management strategies, it is worth reminding that photonic improvements should not degrade the electrical performance of the device. Therefore, a compromise has to be found between photonic and electrical aspects, if one aims at using photon management for improving cells with already high efficiency.

Let us finally remark that most of the works aiming at improving the performance of perovskite solar cells focused on strategies for improved light harvesting. Therefore, the reported improvements concerned mostly the J_SC_ of the device. Although we considered strategies related to single-junction devices, it is worth noting that light harvesting can be boosted by designing tandem structures combining perovskites with other materials such as silicon or CIGS. In contrast with such approaches, from derivations of Shockley –Queisser detailed balance model [67,68], V_OC_ enhancement is also expected upon increasing the external luminescence quantum yield of the device. Perovskite materials show strong radiative recombination at photon energies just below their bandgap, with an internal quantum efficiency that depends on the quality of the perovskite crystals, especially their interfaces [69]. The external luminescence quantum yield of a perovskite solar cell therefore depends of the intrinsic quality of the perovskite material, on parasitic re-absorption in the other layers of the cell [70], and on the structuring of the cell for efficient light extraction [71]. The roles played by these different aspects are inter-dependent. On the one hand, the photon recycling effect recently observed in perovskite solar cells [72] enables a high external luminescence quantum yield (and thus a high V_OC_) in devices with a high intrinsic quality perovskite material and low parasitic re-absorption, without structuring. Photon recycling consists in the re-absorption of the perovskite luminescence by this material itself, followed by re-emission. Therefore, it is a ‘lossless’ mechanism where a photon is re-absorbed and re-emitted until its wave vector is suitable for escaping from the cell. On the other hand, the external luminescence quantum yield can be boosted by structuring, for a lower quality of the perovskite material and/or if parasitic re-absorption occurs.

## Disclosure statement

No potential conflict of interest was reported by the authors.

## Funding

This work was supported by the Natural Science Foundation of Jiangsu Province [grant number BK20171022]; the Scientific Research Foundation; the Scientific Research Foundation for the Returned Overseas Chinese Scholars. Financial support is acknowledged from the Spanish MINECO [Severo Ochoa program, Grant No.: SEV-2015-0522], the MINECO, and the Fondo Europeo de Desarrollo Regional FEDER [Grant No.: MAT2014-52985-R], the Fundació Privada Cellex, and from the EC FP7 Program [Grant No.: ICT-2011.35] under grant agreement No. NMP3-SL-2013-604506.
